# Pharmacogenomic implications of the differential distribution of CYP2C9 metabolic phenotypes among Latin American populations

**DOI:** 10.3389/fphar.2023.1246765

**Published:** 2023-08-11

**Authors:** Guilherme Suarez-Kurtz

**Affiliations:** Coordenação de Pesquisa, Instituto Nacional de Câncer, Rio de Janeiro, Brazil

**Keywords:** biogeographical ancestry, CYP2C9 metabolic phenotypes, Latin American populations, nonsteroidal anti-inflammatory drugs, pharmacogenomic implications

## Abstract

The *CYP2C9* gene encodes the major drug metabolism enzyme CYP2C9. This gene is highly polymorphic, and no-function (CYP2C9*3) plus decreased function (CYP2C9*2, *5, *8 and *11) star alleles (haplotypes) are commonly used to predict CYP2C9 metabolic phenotypes. This study explores the pharmacogenomic implications of the differential distribution of genotype-predicted CYP2C9 phenotypes across Latin American populations. Data from 1,404 individuals from the South American countries Brazil, Colombia and Peru, from Puerto Rico in the Caribbean and from persons with Mexican ancestry living in North America were analysed. The results showed that the distribution of *CYP2C9* alleles and diplotypes, and diplotype-predicted CYP2C9 phenotypes vary significantly across the distinct country cohorts, as well as among self-identified White, Brown and Black Brazilians. Differences in average proportions of biogeographical ancestry across the study groups, especially Native American and African ancestry, are the likely explanation for these results. The differential distribution of genotype-predicted CYP2C9 phenotypes has potentially clinically-relevant pharmacogenomic implications, through its influence on the proportion of individuals at high risk for adverse response to medications that are CYP2C9 substrates, the proportion on individuals with CPIC therapeutic recommendations for dosing and choice of nonsteroidal antinflammatory drugs (NSAIDs) and the number of individuals that need to be genotyped in order to prevent adverse effects of NSAIDs. Collectively, these findings are likely to impact the perceived benefits, cost-effectiveness and clinical adoption of pharmacogenomic screening for drugs that are predominantly metabolized by CYP2C9.

## Introduction

CYP2C9, an enzyme of the cytochrome P450 superfamily, provides the major pathway for metabolism of several commonly-prescribed therapeutic medicines, such as anticoagulants (e.g., warfarin), anticonvulsants (phenytoin), antidepressants (fluoxetine), angiotensin receptor blockers (losartan), diuretics (torsemide) and nonsteroidal anti-inflammatory drugs (NSAIDs). The clinical relevance of the CYP2C9 pathway is reflected in the CPIC (Clinical Pharmacogenetics Implementation Consortium) guidelines: three of the 26 guidelines currently available have therapeutic recommendations based on CYP2C9 metabolic phenotypes, predicted from *CYP2C9* genotypes (Johnson et al., 2017; Theken et al., 2020; Karnes et al., 2021). The *CYP2C9* gene is highly polymorphic, with 85 star alleles (haplotypes) currently defined in the Pharmacogene Variation Consortium (https://www.pharmvar.org/gene/CYP2C9). The frequency distribution of functionally relevant *CYP2C9* alleles listed in the CPIC guidelines shows considerable variability across geographical regions and ethnicities: CYP2C9*2 is most prevalent in Europe and the Middle East, CYP2C9*3 shows the highest frequency among South Asians, while CYP2C9*5,*8 and *11 are typically observed in populations of African descent (Sistonen et al., 2009; Céspedes-Garro et al., 2015; Zhou et al., 2023; https://gnomad.broadinstitute.org). Variation in frequency of functional *CYP2C9* alleles is also observed among the heterogeneous and admixed populations of Latin America, which are the target of the present study. Thus, significant trends for increasing frequency of CYP2C9*2 and CYP2C9*3 as the individual proportion of European ancestry increases, and for increasing frequency of CYP2C9*5 and CYP2C9*11 as African ancestry increases were first reported in an admixed Brazilian cohort (Suarez-Kurtz et al., 2012). Accordingly, both CYP2C9*2 and CYP2C9*3 were reported at lower frequencies in native Tepehuanos from Mexico than in admixed Mexicans (Dorado et al., 2011). Rodrigues-Soares et al. (2020) expanded the study of the distribution of *CYP2C9* variants and predicted phenotypes to 33 populations from Latin America, and concluded that European admixture accounts for the presence of CYP2C9*2 in Native American populations, while CYP2C9*3 was already present in the pre-Columbian Americas.

The present study explores the pharmacogenomic implications of the distinct distribution of clinically relevant *CYP2C9* variants across Latin American populations, represented by Brazilians and the four cohorts of the One Thousand Genomes Project Admixed American (1KG_AMR) superpopulation. The results show that the proportion of individuals at high risk for adverse effects of drugs predominantly metabolized by *CYP2C9*, the proportion of patients with CPIC therapeutic recomendation for adjustment of doses of NSAIDs and the number of patients that need to be genotyped to prevent NSAIDs-associated adverse effects vary markedly among Latin American populations.

## Methods

### Study cohorts

Data from five distinct Latin American cohorts were analyzed in this study: Colombians (denoted CLM, n = 94), Peruvians (PEL, n = 85), Puerto Ricans (PUR, n = 104) and invididuals of Mexican Ancestry (MXL, n = 64) of the Admixed-American superpopulation of the One Thousand Genomes Project (denoted 1KG_AMR; Auton et al., 2015), plus a cohort of adult, healthy, non-related Brazilians (https://www.refargen.org.br/article.php3?id_article=47; n = 1,057), self-identified as White (n = 349), Brown (meaning *Pardo* in Brazilian Portuguese; n = 357) and Black (n = 351), according to the race/Color categories of the Brazilian Census. The geographical origin of the cohorts is shown in Supplementary Figure S1. The term Color and the race/Color categories are capitalized to call attention to their special meaning in the context of the Brazilian Census classification, Color (in Portuguese, “cor”) denoting the Brazilian equivalent of the English term “race”.

The term “cohorts” will be applied to the 1KG_AMR populations and the overall Brazilian sample, while “sub-cohorts” will de used to denote White, Brown and Black Brazilians, and “groups” refer to cohorts and subcohorts. Of note, the data shown for the overall Brazilian cohort were weighed according to the percentages of each race/Color category in the most recent Brazilian Census: White (43.1%), Brown (46.5%) and Black (9.3%) (https://www.ibge.gov.br/en/statistics/social/population/22836-2020-census-censo4.html). Together, these three race/Color groups account for 98.8% of the Brazilian population.

### *CYP2C9* alleles, haplotypes and diplotypes

Five single nucleotide polymorphisms (SNPs) in *CYP2C9* were investigated, namely, the non-functional CYP2C9*3 allele (NC_000010.11:g.94981296A>C, p.I359L, rs1057910) and the decreased-function alleles *2 (NC_000010.11:g.94942290C>T, p.R144C, rs1799853), *5 (NC_000010.11:g.94981301C>G, p.D360E, rs28371686), *8 (NC_000010.11:g.94942309G>A, p.R150H, rs7900194) and *11 (NC_000010.11:g.94981224C>T, p.R335W, rs28371685). Genotype data from the 1KG_AMR groups were retrieved from https://www.ensembl.org/index.html , whereas data for Brazilians were derived from previous studies of the Brazilian Pharmacogenetics Network (Suarez-Kurtz, 2010; Suarez-Kurtz et al., 2012). CYP2C9*1 (reference allele) was assigned by default, i.e., absence of variant alleles at the *CYP2C9* loci interrogated. Individual haplotypes and diplotypes were inferred using the HaploStats software, implemented on the R platform. This software attributes a posterior probability value for the diplotype configuration of each individual on the basis of estimated haplotype frequencies. The minimal posterior probability value for inclusion of an individual in the present analyses was set at 0.95.

### Inference of CYP2C9 metabolic phenotypes and pharmacogenomic implications

PharmGKB gene-specific tables (https://www.pharmgkb.org/page/cyp2c9RefMaterials) were used to assign activity scores (AS) to *CYP2C9* diplotypes (Caudle et al., 2017), for mapping of diplotypes to possible metabolic phenotypes, and for mapping of possible phenotypes to Electronic Health Record (EHR) Priority Result Notation risk (Supplementary Table S1). The assigned phenotypes were also mapped to the therapeutic recommendations’ categories (Strong, Moderate or Optional) of the CPIC guidelines for NSAIDs (Theken et al., 2020).

The procedures described by Tonk et al. (2017) were applied to estimate the number of individuals needed to be genotyped (NNG) in order to prevent one additional adverse event associated with NSAIDs. These procedures are detailed in Supplementary File S1. Briefly, data from a metanalysis by Agúndez et al. (2009) were used to obtain the frequency of gastrointestinal bleeding (denoted *q*) in patients exposed to NSAIDs and the odds ratio (OR) associated with CYP2C9 High Risk metabolic phenotypes. These *q* and OR values plus the combined frequency of the risk-associated CYP2C9 phenotypes in each study group were then entered into the equations described by Tonk et al. (2017) to estimate the NNG metric.

#### Statistical analyses

Chi square tests, available at https://www.icalcu.com/stat/chisqtest.html were applied to assess deviations of genotype distribution from Hardy-Weinberg equilibrium (HWE) and to compare the distribution of alleles, diplotypes and predicted metabolic phenotypes across the study groups. Significance level was set at *p* < 0.05. The Cramér´s V test, an effect size measurement for the chi-square test of Independence, was applied to assess the strength of the association between the study cohorts and either *CYP2C9* Activity Scores or CYP2C9 metabolic phenotypes. The Cramér’s V is calculated as V = √(χ^2^/n*df), where χ^2^: Chi-square statistic, n: total sample number, df: degree of freedom estimated as min(c-1, r-1), in which c: number of colums and r: number or rows. Cramer’s V values obtained were interpreted according to Cohen´s h scale (Cohen, 1988), based on the degrees of freedom (Supplementary File S2).

## Results

### *CYP2C9* alleles and diplotypes

The frequency distribution of *CYP2C9* alleles is shown in Supplementary Table S2. There was no deviation from HWE at any locus. Alleles *5, *8 and *11 were absent or rare (frequency <0.015) in all study groups (cohorts and Brazilian subcohorts) and were combined for the statistical analyses. Allele frequency differed significantly across cohorts (chi square *p =* 0.0003), as well as among the Brazilian subcohorts (*p <* 0.0001). Alleles CYP2C9*2 and *3 accounted for these findings, as their frequency ranged over 5-fold across cohorts and 2-fold in the Brazilian subcohorts: Among cohorts, PEL showed the lowest frequency of both CYP2C9*2 (0.024) and *3 (0.012), while the highest frequency of *2 (0.139) and *3 (0.064) were found in PUR and CLM, respectively. In Brazilians, both CYP2C9*2 and *3 were less common in self-reported Black than in White or Brown individuals.

Table 1 shows the frequency of inferred *CYP2C9* diplotypes in the study groups. Due to their absence of rarity (frequency <0.005) in all groups, diplotypes formed by the reference CYP2C9*1 and one of *5, *8 or *11 alleles were combined for the statistical analyses. Six diplotypes were then identitied, and their distribution differed significantly among cohorts (*p* = 0.006) and subcohorts (*p* = 0.0004). Reference homozygotes (CYP2C9*1/*1) ranged in frequency between 0.638 (PUR) and 0.929 (PEL) across cohorts, and between 0.670 (White) and 0.812 (Black) in the Brazilian subcohorts. CYP2C9*1/*2 was the most common diplotype containing a variant allele, ranging in frequency from 0.047 (PEL) to 0.223 (PUR) across cohorts, and between 0.103 (Black) and 0.195 (White) in Brazilians. CYP2C9*1/*3 ranged in frequency from 0.024 (PEL) to 0.106 (CLM) across cohorts and between 0.040–0.093 in Brazilians. CYP2C9*2/*2 was absent in CLM, MXL and PEL, and present in <2% of PUR and Brazilians, while *3 homozygosis was not found. CYP2C9*2/*3 compound heterozygotes were not detected in MXL and PEL, and ranged in frequency between 0.01–0.02 in the other cohorts and subcohorts.

**TABLE 1:** Distribution of *CYP2C9* diplotypes in Latin American cohorts.

		CYP2C9 genotypes						Chi -square *p*-value
	n	*1/*1	*1/*2	*1/*3	*2/*2	*2/*3	*1/var^a^	
Cohorts								
Brazilian	1,057	0.707	0.157	0.082	0.017	0.014	0.021	0.006
CLM^b^	94	0.649	0.223	0.106	0	0.021	0	
MXL	64	0.750	0.203	0.047	0	0	0	
PEL	85	0.929	0.047	0.024	0	0	0	
PUR	104	0.635	0.229	0.076	0.019	0.010	0.029	
Brazilian subcohorts								
Black	351	0.812	0.103	0.040	0.003	0.009	0.034	0.0004
Brown	357	0.725	0.132	0.093	0.017	0.008	0.022	
White	349	0.670	0.195	0.077	0.020	0.020	0.017	

n = number of individuals.

^a^ Comprises CYP2C9*1/*5, *1/*8 and *1/*11.

^b^ CLM, colombians; MXL, invididuals of Mexican Ancestry; PEL, peruvians; PUR, puerto ricans, from the 1 KG, project.

### Activity scores and predicted CYP2C9 metabolic phenotypes

**FIGURE 1:**
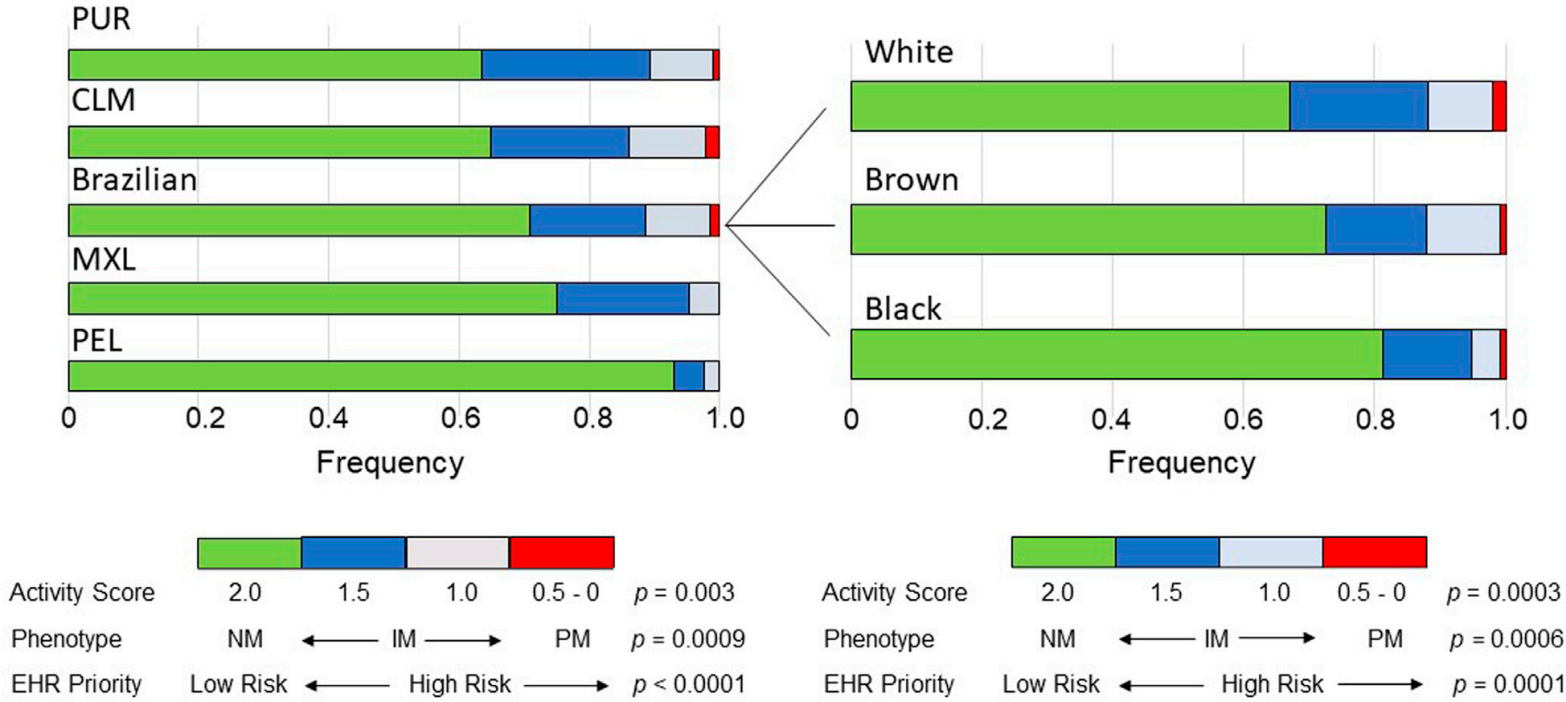
Bar plots of the distribution across the study groups of the Activity Scores of *CYP2C9* diplotypes, predicted CYP2C9 metabolic phenotypes and EHR Priority Notation Risk of the predicted phenotypes. Data used to construct the plots are presented in Supplementary Table S3. The *p* values refer to chi-square tests across cohorts or Brazilian subcohorts. NM, normal metabolizer; IM, intermediate metabolizer, PM, poor metabolizer.

The distribution of AS´s assigned to the *CYP2C9* diplotypes differed significantly and largely across cohorts (*p* = 0.003) and Brazilian subcohorts (*p* = 0.0003; Supplementary Table S3 and Figure 1). The widest frequency range was observed for AS = 1.0 (5-fold in both cohorts and subcohorts) and AS = 1.5 (1.6-fold in cohorts and 2.6-fold in subcohorts), while AS = 0 was not observed, AS = 0.5 was absent in MEX and PEL but present in 1%–2% of the other groups and AS = 2.0 ranged in frequency from 0.635 (PUR) to 0.929 (PEL) among cohorts and from 0.670 (White) to 0.812 (Black) in Brazilians.

As anticipated from the distribution of the AS´s of the *CYP2C9* diplotypes, the frequency of predicted CYP2C9 metabolic phenotypes varied significantly across cohorts (*p* = 0.0007) and subcohorts (*p* = 0.0006): the IM phenotype (AS = 1.0 and 1.5) showed the largest range of frequencies in cohorts (5-fold) and subcohorts (1.7-fold), while PMs and NMs ranged in frequency as reported above for AS = 0.5 and AS = 2.0, respectively (Supplementary Table S3 and Figure 1). Despite the highly significant diferences in the frequency distribution of AS´s and CYP2C9 phenotypes across the study groups, the Cramer´s V test pointed to small effect sizes in the cohorts and Brazilian subcohorts (Supplementary Table S3).

### Pharmacogenomic implications

For a first assessement of the pharmacogenomic implications of the differential distribution of CYP2C9 phenotypes across Latin American populations, the PharmGKB CYP2C9 Example CDS Table (https://www.pharmgkb.org/page/cyp2c9RefMaterials) was used to dichotomize phenotypes to either Normal/Routine/Low Risk (NM phenotype, AS = 2) or Abnormal/Priority/High Risk (IM and PM phenotypes, AS = 0–1.5). The proportion of High Risk phenotypes ranged 5.2-fold across cohorts (*p* < 0.0001) with PEL having the lowest proportion (7.1%) and PUR, the highest (36.5%); in the Brazilian subcohorts the proportion of High Risk ranged 1.8-fold (*p* = 0.0001), decreasing progressively from White to Brown and then to Black individuals (Figure 1).

Next, the CYP2C9 phenotypes were mapped to the classification of therapeutic recommendations (Strong, Moderate or Optional) in the CPIC guidelines for NSAIDs (Theken et al., 2020). Recommendation to “Initiate therapy with the recommended starting dose” of NSAIDS is classified as Strong for NMs and Moderate for IMs with AS = 1.5. For IMs with AS = 1.0 and PMs, the CPIC recommendations and their classification differ for the various NSAIDs. For AS = 1.0, recommendations are for reduction of the initial dose of meloxicam (Moderate), choice of alternative drug to piroxicam (Moderate) and tenoxicam (Optional), or initiate therapy with the lowest recommended dose of other NSAIDs (Moderate). For PMs, CPIC guidelines recommend alternative drugs to meloxicam (Moderate), piroxicam (Moderate) or tenoxicam (Optional), and reduced doses of other NSAIDs (Moderate). Collectively, Moderate or Optional recommendations for adjustment of the initial dose or choice of alternative NSAIDs apply to 2.4%–13.8% of individuals in the study cohorts and to 5.1%–12.0% in the Brazilian subcohorts (Table 2). A similar percentagem of individuals may require adjusting dose or drug choice for other medications that are mainly metabolized by CYP2C9.

**TABLE 2:** Classification of CPIC therapeutic recommendations for NSAIDs^a^.

		Usual starting dose		Adjust initial dose
	n	Strong (AS = 2)	Moderate (AS = 1.5)	Moderate/Optional (AS 0–1.0)^b^
Cohorts				
Brazilian	1,057	0.707	0.178	0.115
CLM^c^	94	0.649	0.213	0.138
MXL	64	0.750	0.203	0.047
PEL	85	0.929	0.047	0.024
PUR	104	0.635	0.260	0.106
Brazilian subcohorts				
Black	351	0.812	0.137	0.051
Brown	357	0.725	0.154	0.120
White	349	0.670	0.212	0.117

^a^ Data show proportion of individuals for the CPIC therapeutic recommendations (Theken et al., 2020).

^b^ Optional for tenoxicam, Moderate for other NSAIDs.

^c^ CLM, Colombians; MXL, invididuals of Mexican Ancestry; PEL, Peruvians; PUR, Puerto-Ricans, from the 1 KG, project.

**FIGURE 2:**
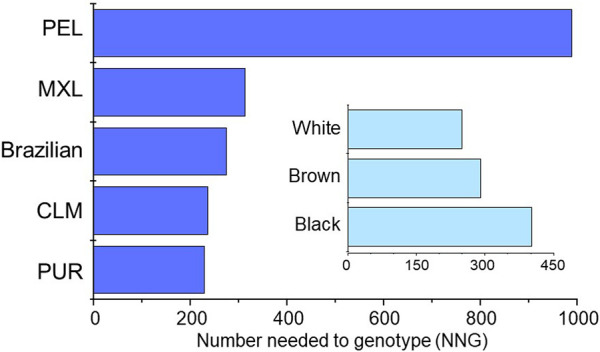
Plots of the estimated NNG to avoid one additional event of gastrointestinal bleeding associated with NSAIDs therapy in the study groups. The procedures used to estimate the NNG´s are described in Supplementary File S1.

Finally, the number of individuals needed to be genotyped (NNG) in order to prevent one additional adverse event induced by NSAIDs were estimated as described in Supplementary File S1. Based on data from a metanalysis of the risk of gastrointestinal bleeding in patients exposed to NSAIDs Agúndez et al. (2009) the estimated NNG to avoid one additional bleeding event ranged from 230 (PUR) to 989 (PEL) among cohorts, and from 250 (White) to 402 (Black) in Brazilians (Figure 2).

## Discussion

The present study analysed the distribution of *CYP2C9* functional variants and predicted CYP2C9 metabolic phenotypes among Latin American populations and explored its phamacogenomic implications. Latin America comprises 20 countries and six territories distributed over 20 million km^2^ across South, Central and North America, and the Caribbean. The extent and dynamics of admixture among the three major parental populations, namely, autochthonous (Native) Amerindians, Europeans and sub-Saharan Africans, resulted in the kaleidoscopic diversity of the present-day Latin American population, in excess of 650 million people (∼9% of global population). Importantly, the relative proportions of these major ancestral roots vary widely across and within nations in the American continent (Pena et al., 2011; Bonifaz-Peña et al., 2014; Ruiz-Linares et al., 2014; Belbin et al., 2018; Suarez-Kurtz and Parra, 2018), and the resulting heterogeneity represents a major caveat against lumping Latin Americans into a single racial*/*ethnic category, such as Latino*/*Hispanic (Huddart et al., 2019).

A total of 1,404 samples from the South American countries Brazil, Colombia and Peru, from Puerto Rico in the Caribbean and from individuals with Mexican ancestry living in North America were analysed in this study. The results showed that the distribution of *CYP2C9* alleles and diplotypes, and diplotype-predicted CYP2C9 phenotypes vary significanly across the distinct cohorts, as well as among self-identified as White, Brown or Black Brazilians. Differences in average proportions of biogeographical ancestry across the study groups (Supplementary Table S4), especially Native American and African ancestry, are the likely explanation for these results. Previous studies of Native Americans and admixed cohorts of predominant Native ancestry revealed absence or rarity of all variant *CYP2C9* alleles analysed here. Examples are the absence of CYP2C9*3, *5, *8 and *11, and rarity of *2 (frequency 0.01) in Native American samples of the Human Genome Diversity Project (Cavali-Sforza, 2005); absence of CYP2C9*3, *5 and *11, and rarity of CYP2C9*2 (frequency 0.01) in Guarani from an indigenous reservation area in Brazil (Vargens et al., 2012); absence of CYP2C9*2 and *3 in nine and seven out of fifteen Ibero-American populations with estimated average Native ancestry >85% (Rodrigues-Soares et al., 2020). Accordingly, PEL, with the highest proportion of Native ancestry (0.84) among this study cohorts, displayed the lowest frequency of alleles CYP2C9*2 (0.024) and *3 (0.012), and absence of alleles *5, *8 and *11. By contrast, the latter three alleles, which associate with African descent were detected only in Brazilians and PUR, the two study cohorts with the highest average proportions of African ancestry. Among Brazilian subcohorts, the frequency of CYP2C9*8 increased from self-reported White, to Brown to Black iindividuals, in parallel with the average proportions of African ancestry (Supplementary Table S4).

A previous study of genetic divergence across Latin American populations, using the fixation index statistics (F_*ST*_), reported little pharmacogenetic differentiation for the *CYP2C9* variant alleles examined in the present analysis (Suarez-Kurtz and de Araújo, 2022). In addition, the Cramér´s V test disclosed small magnitude of the effects of association between the study groups (cohorts and Brazilian subcohorts) and AS´s or metabolic phenotypes. Nevertheless, the distinct distribution of genotype-predicted CYP2C9 phenotypes across the study groups resulted in potentially relevant pharmacogenomic implications. First, the proportion of individuals at high risk for “an adverse response to medications that are affected by CYP2C9”, according to the PharmGKB EHR Priority Result Notation (https://www.pharmgkb.org/page/cyp2c9RefMaterials), differed significantly across study cohorts and subcohorts. Second, the proportion on individuals with CPIC therapeutic recommendations for initial dosing and/or choice of NSAIDs (Theken et al., 2020) ranged 5.4-fold across cohorts and 2.4-fold in the Brazilian subcohorts. Importantly, these CPIC recommendations are classified as Moderate or, in the case of tenoxicam, Optional. There are no Strong recommendations for dose adjustment or choice of a different drug in the NSAIDs CPIC guideline, although a Strong recommendation for dose adjustments in CYP2C9 PMs is made in the CPIC guideline for phenytoin (Karnes et al., 2021); such recommendation applies to 1% – 2% of Brazilians, CLM and PUR, but not to MXL or PEL, due to the absence of PMs in the latter two cohorts. Of notice, the CPIC recommendation for phenytoin dosing rely also on the presence/absence of the HLA-B*15:02 haplotype. Thirdly, the NNG in order to prevent one additional gastrointestinal bleeding event in patients exposed to NSAIDs ranged 4.3-fold (230–989 individuals) across cohorts and 1.6-fold (250–402) in Brazilian subcohorts. Collectively, these findings are likely to impact the perceived benefits, cost-effectiveness and clinical adoption of pharmacogenetic screening for NSAIDs and other drugs that are CYP2C9 substrates. Indeed, the probability that a patient will have a clinically relevant pharmacogenetic variant is a key parameter in cost-effectiveness analyses of routine PGx screening (Veenstra, 2016; Hughes, 2018).

Although a thorough discussion of cost-effectiveness is out of scope here, an estimate of the costs of *CYP2C9* genotyping to prevent one additional NSAID-associated gastrointestinal bleeding event in the cohorts might be useful to put the NNG into context. Using the estimated NNGs and the current US$ vs. Brazilian Real exchange rate, the purchase cost of reagents and disposables for DNA extraction and genotyping the five target *CYP2C9* variants, using Taqman allele discrimination assays, ranges from US$ 5,131 (PUR) to 22,031 (PEL), when 10 DNA samples are assayed concomitantly. Among Brazilians the corresponding costs range from US$ 5,571 (White) to 8,945 (Black).

Limitations of this study include: First, *CYP2C9* genotyping was restricted to the most common and decreased function alleles listed in the CPIC guidelines for CYP2C9 substrates, and the reference *1 allele was assigned by default (Johnson et al., 2017; Theken et al., 2020; Karnes et al., 2021). Second, the cohorts investigated do not fully represent the diversity and heterogeneity of Latin American populations, and caution must be exerted in extrapolating the present findings across Latin America. The evidence (Lewontin, 1973) that the largest proportion of genetic variation (*>*80%) among human populations is found within local geographic groups is especially relevant in highly admixed populations, such as Latin Americans. Accordingly, the absence of genetic ancestry analysis adds to the limitations of this study. Finally, the NNG estimates were based on odd ratios and frequency of adverse events in trials carried out in distinct populations and clinical settings, which may not be reproduced in the cohorts examined in this study.

In conclusion, the present analyses show that the differential distribution of CYP2C9 functional alleles across Latin American populations has pharmacogenomic implications that are likely to impact the perceived benefits, cost-effectiveness and clinical adoption of pharmacogenomic screening for drugs that are predominantly metabolized by CYP2C9. The diversity and heterogeneity of Latin Americans should caution against their lumping into a single racial*/*ethnic category, such as Latino*/*Hispanic, as is often done in pharmacogenomic trials.

## Data Availability

The original contributions presented in the study are included in the article/Supplementary Material, further inquiries can be directed to the corresponding author.
