# Supplementary figures and images for: Pharmacogenomic implications of the differential distribution of CYP2C9 metabolic phenotypes among Latin American populations
Source: Front Pharmacol. 2023 Aug 11;14:1246765. doi: 10.3389/fphar.2023.1246765 (PMC10488705; doi:10.3389/fphar.2023.1246765)

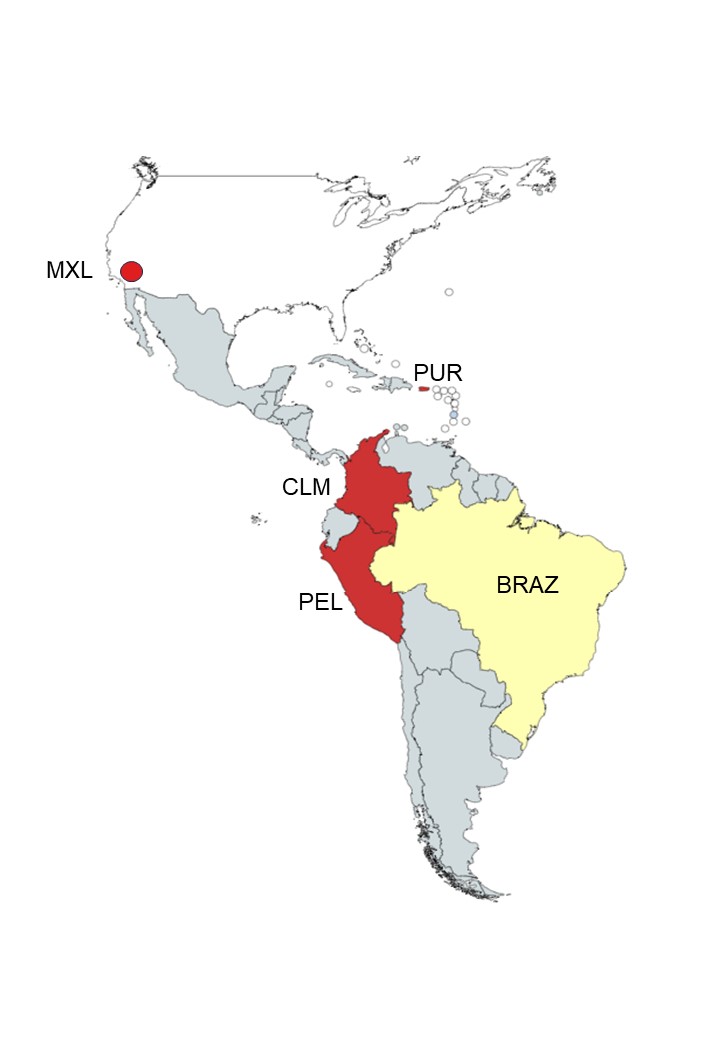

Supplement: Supplementary file 1 [file Image1.JPEG]
