# Supplementary material for: Pharmacogenomic implications of the differential distribution of CYP2C9 metabolic phenotypes among Latin American populations
Source: Front Pharmacol. 2023 Aug 11;14:1246765. doi: 10.3389/fphar.2023.1246765 (PMC10488705; doi:10.3389/fphar.2023.1246765)
Supplement: Supplementary file 2 [file DataSheet1.pdf]

Supplementary Table 1. Mapping of *CYP2C9* diplotypes to CYP2C9 phenotypes and clinical risk [#]

***CYP2C9* Diplotype**	**Activity Score**	**Inferred Phenotype**	**EHR Priority Notation**
**1/*1*	2.0	Normal Metabolizer (NM)	Normal/Routine/Low Risk
**1/*2*	1.5	Intermediate Metabolizer (IM)	Abnormal/Priority/High Risk
**1/*3*	1.0	Intermediate Metabolizer (IM)	Abnormal/Priority/High Risk
**1/*5*	1.5	Intermediate Metabolizer (IM)	Abnormal/Priority/High Risk
**1/*8*	1.5	Intermediate Metabolizer (IM)	Abnormal/Priority/High Risk
**1/*11*	1.5	Intermediate Metabolizer (IM)	Abnormal/Priority/High Risk
**2/*2*	1.0	Intermediate Metabolizer (IM)	Abnormal/Priority/High Risk
**2/*3*	0.5	Poor Metabolizer (PM)	Abnormal/Priority/High Risk
**3/*3*	0.0	Poor Metabolizer (PM)	Abnormal/Priority/High Risk

[#] From PharmGKB gene-specific information tables (https://www.pharmgkb.org/page/cyp2c9RefMaterials).

Supplementary Table 2. Distribution of *CYP2C9* SNPs in Latin American cohorts

		**1*	**2*	**3*	**5*	**8*	**11*	Chi -square
	n	default	rs1799853	rs1057910	rs28371686	rs7900194	rs28371685	*P* value
**Cohorts**								
Brazilian	1057	0.840	0.092	0.053	0.002	0.003	0.010	
CLM#	94	0.814	0.122	0.064	0	0	0	
MXL	64	0.875	0.102	0.023	0	0	0	0.0002
PEL	85	0.964	0.024	0.012	0	0	0	
PUR	104	0.803	0.139	0.043	0.005	0.005	0.005	
**Brazilian subcohorts**								
Black	351	0.900	0.058	0.024	0.001	0.014	0.002	
Brown	357	0.851	0.087	0.051	0.002	0.007	0.002	0.0002
White	349	0.815	0.128	0.049	0.002	0.003	0.003	

n = number of individuals. # CLM, Colombians; MXL, invididuals of Mexican Ancestry; PEL, Peruvians; PUR, Puerto Ricans, from the 1KG Project.

Supplementary Table 3. Distribution CYP2C9 activity scores and metabolic phenotypes

		Activity Scores				Chi -square	CYP2C9 phenotypes			Chi -square
	n	2	1.5	1.0	0.5		NM	IM	PM	
**Cohorts**										
Brazilian	1057	0.707	0.178	0.106	0.009	*P* = 0.003	0.707	0.284	0.009	*P* = 0.0007
CLM[#]	94	0.649	0.213	0.117	0.021	V = 0.065	0.649	0.330	0.021	V = 0.095
MXL	64	0.750	0.203	0.047	0		0.750	0.250	0	
PEL	85	0.929	0.047	0.024	0		0.929	0.071	0	
PUR	104	0.635	0.260	0.096	0.010		0.635	0.356	0.010	
**Brazilian subcohorts**										
Black	351	0.812	0.137	0.043	0.009	*P* = 0.0003	0.812	0.179	0.009	*P* =0.0006
Brown	357	0.725	0.154	0.112	0.008	V = 0.065	0.725	0.266	0.008	V = 0.098
White	349	0.670	0.212	0.097	0.020		0.670	0.309	0.020	

n = number of individuals, NM = normal metabolizer, IM = intermediate metabolizer, PM = poor metabolizer.

[#] CLM, Colombians; MXL, invididuals of Mexican Ancestry; PEL, Peruvians; PUR, Puerto Ricans, from the 1KG Project.

V, Cramer´s V value for effect size of the association of study groups (cohorts and subcohorts) with Activity Scores or CYP2C9 phenotypes

Supplementary Table 4. Average biogeographical ancestry of study groups[#]

	Native	African	European
**Cohorts**			
Brazilians	0.12 (0.10-0.14)	0.20 (0.18-0.22)	0.68 (0.66-0.70)
CLM[##]	0.24 (0.19-0.29)	0.06 (0.05-0.07)	0.70 (0.56-0.84)
MEX	0.53 (0.40-0.66)	0.02 (0.01-0.02)	0.45 (0.34-0.56)
PEL	0.84 (0.66-1.02)	0.02 (0.02-0.03)	0.14 (0.11-0.16)
PUR	0.10 (0.08-0.11)	0.11 (0.09-0.13)	0.80 (0.65-0.95)
**Subcohorts**			
Black	0.12 (0.10-0.13)	0.42 (0.39-0.46)	0.46 (0.43-0.49)
Brown	0.13 (0.11-0.15)	0.25 (0.22-0.28)	0.62 (0.59-0.65)
White	0.10 (0.08-011)	0.10 (0.08-012)	0.80 (0.78-0.83)

[#] Mean (CI95%). [##] CLM, Colombians; MXL, invididuals of Mexican Ancestry; PEL, Peruvians; PUR, Puerto Ricans, from the 1KG Project.
Data for CLM, MXL, PEL and PUR from Debortoli et al. (2021); data for Brazilians from Pena et al., (2012).

References

Debortoli G, de Araujo GS, Fortes-Lima C, Parra EJ, Suarez-Kurtz G. (2021). Identification of ancestry proportions in admixed groups across the Americas using clinical pharmacogenomic SNP panels. *Sci Rep.* 2021 Jan 13;11(1):1007. doi: 10.1038/s41598-020-80389-9.

Pena SD, Di Pietro G, Fuchshuber-Moraes M, Genro JP, Hutz MH, Kehdy Fde S et al. (2011). The genomic ancestry of individuals from different geographical regions of Brazil is more uniform than expected. *PLoS One.* Feb 16;6(2):e17063. doi: 10.1371/journal.pone.0017063.

Supplementary File 1

This supplementary file presents the procedures described by Tonk et al. [2017] to estimate the number of individuals who need to be genotyped (NNG) in order to prevent one additional adverse event to a drug. Briefly, the odds ratio (OR) of pharmacogenetic (PGx) associations with drug-induced adverse reactions, the frequency of the adverse reactions (*q*) and the frequency of the PGx variant(s) associated with the adverse reaction (*p*) are entered into equations (1 – 4) below, to obtain the values of the cells *a-d* in a 2 x 2 contingency table:

		Adverse reaction		
		Affected	Unaffected	Total
**PGx variant(s)**	Present	*a*	*b*	*p*
	Absent	*c*	*d*	*(1-p)*
	Total	*q*	*(1-q)*	*1*

$$a = \frac{(((p\times OR+(1-p)+q\times(OR-1))-\sqrt{(((-p\times OR-(1-p)-q\times(OR-1))^2-4\times(OR-1)\times p\times q\times OR)}))}{(2\times(OR-1))} \quad (1)$$

$$b = p - a \quad (2)$$

$$c = q - a \quad (3)$$

$$d = 1 - a - b - c \quad (4)$$

Next, the risk difference (RD) and NNG are calculated using equations 5 and 6:

$$\mathrm{RD} = \frac{a}{a+b} - \frac{c}{c+d} \quad (5)$$

$$\mathrm{NNG} = \frac{1}{(\mathrm{RD}{*}p)} \quad (6)$$

For the present analyses, the OR (1.78) was derived from Agúndez et al., (2009), *q* was set at 2% and the *p* values represent the combined frequency of the CYP2C9 metabolic phenotypes associated with gastrointestinal bleeding in the index study, namely IM and PM.

Cohorts	OR	*q*	*p*	NNG
Brazilians	1.78	0.02	0.293	275
CLM[#]	1.78	0.02	0.351	238
MXL	1.78	0.02	0.250	314
PEL	1.78	0.02	0.071	989
PUR	1.78	0.02	0.365	230
Brazilian subcohorts				
Black	1.78	0.02	0.188	402
Brown	1.78	0.02	0.279	291
White	1.78	0.02	0.330	250

[#] CLM, Colombians; MXL, invididuals of Mexican Ancestry; PEL, Peruvians; PUR, Puerto Ricans, from the 1KG Project.

References

Agúndez JA, García-Martín E, Martínez C. (2009). Genetically based impairment in CYP2C8- and CYP2C9-dependent NSAID metabolism as a risk factor for gastrointestinal bleeding: is a combination of pharmacogenomics and metabolomics required to improve personalized medicine? *Expert Opin Drug Metab Toxicol*. 5, 607-620. doi: 10.1517/17425250902970998.

Tonk ECM, Gurwitz D, Maitland-van der Zee AH, Janssens ACJW. (2017). Assessment of pharmacogenetic tests: presenting measures of clinical validity and potential population impact in association studies. *Pharmacogenomics J.* 17, 386-392. doi: 10.1038/tpj.2016.34.

Supplementary File 2: Interpretation of Cramér´s V, using Cohen´s h scale (Cohen, 1988)

Degrees of	Effect size			
freedom	negligible	small	medium	large
1	0 < .10	.10 < .30	.30 < .50	.50 or more
2	0 < .07	.07 < .21	.21 < .35	.35 or more
3	0 < .06	.06 < .17	.17 < .29	.29 or more
4	0 < .05	.05 < .15	.15 < .25	.25 or more
5	0 < .05	.05 < .13	.13 < .22	.22 or more

Reference

Cohen, J. (1988). Statistical Power Analysis for the Behavioral Sciences (2nd ed.). Hillsdale, NJ: Lawrence Erlbaum Associates, Publishers.
